# Evidence-Informed Surgical Systems Strengthening with Meaningful Stakeholder Involvement in Low-Resource Settings: A Response to Recent Commentaries

**DOI:** 10.34172/ijhpm.2023.8387

**Published:** 2024-01-14

**Authors:** Leon Bijlmakers, Ruairí Brugha, Martilord Ifeanyichi, Jakub Gajewski, Henk Broekhuizen

**Affiliations:** ^1^Radboud University Medical Centre, Nijmegen, The Netherlands; ^2^Institute of Global Surgery, RCSI University of Medicine and Health Sciences, Dublin, Ireland; ^3^Global Surgery Policy Unit, LSE Health, London School of Economics and Political Science, London, UK; ^4^Health Policy Research Group, Department of Pharmacology and Therapeutics, College of Medicine, University of Nigeria Enugu Campus, Enugu, Nigeria; ^5^Centre for Global Surgery, University of Stellenbosch, Cape Town, South Africa

 We welcome the commentaries published by the *International Journal of Health Policy and Management* on Broekhuizen and colleagues’ paper entitled “Improving access to surgery through surgical team mentoring – policy lessons from group model building with local stakeholders in Malawi.”^1^ All five commentaries support the methodology employed — mixed methods approach, combining stakeholder input obtained through group model building (GMB) workshops, plus follow-up consultations and dynamic modelling. They also acknowledge the potential application of the findings, the transfer of lessons learned and proposed scenarios for surgical team mentoring, along with the associated policy implications, in other settings, for example South Africa.^2^ Moreno et al go as far as suggesting that the mentoring model evaluated in the SURG-Africa project in Malawi may serve as an example for other specialities (ie, family medicine in Colombia).^3^

 Several commentaries outline the elements required to make surgical team mentoring work in practice: for example, strengthening the entire surgical ecosystem,^2^ defining district hospital surgical service packages,^2^ clarifying the value of surgical task-shifting,^4^ covering the additional cost of increased surgical output,^5,6^ overcoming disincentives to surgical mentoring,^5^ and appropriate governance and regulatory processes so as to ensure quality and accountability.^5^

 We support Hanna’s summation, calling for “a common framework” for the articulation, design and reporting (monitoring) of surgical system strengthening interventions. He argues that situation analysis-informed development of national surgical, obstetric and anaesthesia plans (NSOAPs) has not happened in many countries, and suggests that “there are even fewer published examples of NSOAP-driven … policy interventions” that could resolve “system vulnerability.” In contrast, though, and contrary to our own experience, Henry argues that strategic implementation frameworks are not all that important as long as stakeholders who promote equitable access to surgery employ “system-based thinking coupled with implementation science.”^6^ We have difficulty seeing how that would work out in the actual practice of health priority setting and allocating scarce resources, both at national and sub-national (district) levels.

 Surgery cannot be seen in isolation from other competing health priorities, let alone non-health priorities. Conceptual frameworks and practical guidelines, for example for health benefit package design (or revision), go a long way in assisting national health policy-makers in making evidence-informed decisions, whereby genuine stakeholder deliberation and transparency are considered key principles.^7,8^ Such processes are increasingly being implemented in a range of countries, and typically require health technology assessment expertise. They also require a suitable approach to assess, appraise and prioritise health interventions on the basis of evidence around multiple, preferably predefined criteria, such as efficacy of interventions, cost-effectiveness, burden of diseases that can be averted, and budget impact. This can be challenging in a resource-limited context as the required evidence may be at best partial or not available at all, causing uncertainty that needs to be managed.^9^ Such challenges make local stakeholder involvement even more important, because decisions to reallocate scarce resources may have consequences that can easily be overlooked. One can think of changes in patients’ care seeking behaviour or patient referral patterns, changes in workload, or changed requirements for medical supplies. Involving stakeholders with operational service delivery experience in network analysis and the creation of causal loop diagrams may also allow the identification of new leverage points, although a recent paper warns against erroneous cause-and-effect conclusions if no proper quantitative or qualitative approaches are applied.^10^

 Overall, the commentaries underscore the great potential of implementation research in support of surgical, obstetric and anaesthesia (SOA) systems strengthening, and the need for high-level policy commitment. Noteworthy developments since our paper was e-published, in August 2021, include:

- With the adoption of resolution WHA76.2 by the 76th World Health Assembly in May 2023, World Health Organization (WHO) made a powerful call on member states to create national policies for sustainable funding, effective governance and universal access to needs-based emergency, critical and operative care.^11^
- Several implementation research-based articles have been published on systemic barriers to and enablers of universal surgical service coverage. These include papers from the SURG-Africa project, one of which also used the technique of GMB, this time in Zambia, combining it with modelling to explore policy options for embedding surgical team mentoring into existing policies.^12^ Another paper used network and complexity theory to analyse the functionality of surgical referral systems in low-resource settings.^13^ We also published a paper demonstrating the successful participatory research model for collaboration in three African countries between a wide range of in-country stakeholders, local ministries of health and other regulatory bodies, as part of the international SURG-Africa research consortium.^14^
- Namibia and Zimbabwe have developed and launched NSOAPs, joining countries that already had such plans in place (Ethiopia, Senegal, Zambia, Tanzania, Rwanda, Nigeria, and Madagascar).^15,16^ The delegates from 40 African countries at the (maiden) Pan-African Surgical Healthcare Forum, in July 2023, unanimously agreed that all African countries would need to expedite the development of national surgical plans. However, the scaling up of surgical care through the development of stand-alone national surgical plans, separate from mainstream national health strategic planning and budgeting, is questionable. It is time to evaluate if the scale of investment and effort required for NSOAP development is warranted, and consider postponement of further national replications until there is sufficient evidence from evaluations of existing plans that they achieve their aims.
- Other complementary approaches can help raise essential surgery on national health priority rankings. Several countries have embarked on defining national health service packages, also dubbed “health benefit packages,” which include SOA care or aspects thereof. For example, as part of its national universal health coverage benefit package design, the government of Pakistan developed a district essential package of health services delivered by public and private sector facilities at three delivery platforms (community level, primary healthcare centres, and first-level hospitals). Evidence was gathered and reviewed in respect of burden of disease, intervention quality and uptake, feasibility, cost-effectiveness, budget impact, financial protection, and equity implications for four clusters of conditions. One of these clusters was reproductive, maternal, new-born, child, and adolescent healthcare, including obstetrics; another cluster was non-communicable diseases and injury prevention, including basic surgery.^17,18^ In Rwanda, the Ministry of Health together with the Rwanda Social Security Board, the National University of Rwanda, and the country’s Palliative Care Association, with support from WHO and other external partners, engaged in a consultative process to develop an oncology services benefit package which includes multiple surgical procedures. Once adopted this package will guide future resource allocation and reimbursement decisions.

Assessing whether the above efforts and initiatives are successful, in terms of their actual contribution to achieving universal access to quality SOA care, remains a challenge, and calls for further implementation research of sufficient methodological rigour, linking it to local policy processes and programme implementation, and involving local stakeholders in a meaningful manner.

 There is no one “best approach” for involving and building the support of stakeholders for surgical systems strengthening, although explicitly taking a systems dynamics perspective and undertaking GMB have clear advantages through involving senior clinicians with technical expertise, alongside policy-makers with a remit for national decision-making. In light of the need to coordinate and invest in multi-systems level strengthening, it is equally important to involve district surgical clinicians who are the first point of care for delivering essential emergency and elective surgery to neglected rural populations. The 23 workshop participants in our study in Malawi included representatives from the Directorate of Clinical Services of the Ministry of Health, surgical mentors from two central hospitals who supported district surgical clinicians and managed surgical referrals, and clinical staff from several district hospitals who had received surgical mentoring visits.

## Ethical issues

 Not applicable.

## Competing interests

 Authors declare that they have no competing interests.
